# Pentoxifylline with metformin treatment improves biochemical parameters in patients with nonalcoholic steatohepatitis

**DOI:** 10.2478/jomb-2019-0043

**Published:** 2020-09-02

**Authors:** Milica Ćulafić, Sandra Vezmar-Kovačević, Violeta Dopsaj, Branislav Oluić, Nemanja Bidžić, Branislava Miljković, Đorđe Ćulafić

**Affiliations:** 1 University of Belgrade, Faculty of Pharmacy, Department of Pharmacokinetics and Clinical Pharmacy; 2 University of Belgrade, Faculty of Pharmacy, Clinical Centre of Serbia, Department of Medical Biochemistry, Belgrade; 3 Clinical Center of Serbia, Emergency Center, Belgrade; 4 Clinical Center of Serbia, Clinic for Digestive Surgery, Belgrade; 5 University of Belgrade, School of Medicine, Clinical Center of Serbia, Clinic for Gastroenterology and Hepatology

**Keywords:** Nash, pentoxifylline, metformin, treatment, steatohepatitis, Nash, pentoksifilin, metformin, terapija, steatohepatitis

## Abstract

**Background:**

The progression of the nonalcoholic fatty liver disease to nonalcoholic steatohepatitis (NASH) is multifactorial, and there is still a lack of approved medications for its treatment. The study aimed to evaluate the impact of combined treatment with Pentoxifylline and Metformin on biochemical parameters in patients with Nash. Setting: Outpatient hepatology clinic.

**Methods:**

A prospective trial was conducted. The first cohort included patients with biopsy-proven Nash, while the second cohort consisted of patients with biopsy-confirmed NAFLD. Blood tests were checked at baseline and every three months. Pentoxifylline at a dosage of 400 mg t.i.d. and Metformin at the dosage of 500 mg t.i.d. were introduced for six months in Nash group. The impact of the treatment was assessed based on biochemical results after combined treatment with low-cost medications.

**Results:**

All 33 Nash patients completed 24 weeks of treatment. We observed significant improvement (p<0.05) of median values after treatment for the following parameters: serum uric acid levels decreased by 51.0 mmol/L, calcium decreased for 0.27 mmoL/L, magnesium showed an increase of 0.11 mmoL/L. Insulin resistance improved as a reduction of HOMA - IR by 1.3 was detected. A significant decrease of median in liver enzymes, alanine aminotransferase, aspartate aminotransferase and gamma-glutamyltransferase by 24.0 U/L, 9.1 U/L, 10.8 U/L respectively, was noted.

**Conclusions:**

Pentoxifylline and Metformin may provide possible treatment option in Nash. Some new potential benefit of the therapy in improving liver function whilst decreasing cardiovascular risk was perceived.

## Abstract (sr)

**Uvod:**

Progresija nealkoholne masne bolesti jetre (NAFLD) do nealkoholnog steatohepatitisa (NASH) je multifaktorijalna, i još uvek ne postoje zvanično odobreni lekovi za terapiju. U studiji je procenjivan uticaj kombinovane terapije pentoksifilinom i metforminom na biohemijske parametre kod pacijenata sa NASHom. Okruženje: Hepatološka ambulanta klinike.

**Metode:**

Studija je bila prospektivnog karaktera. Prva grupa uključila je pacijente sa biopsijom potvrđenim NASHom, dok su drugu grupu činili pacijenti sa biopsijom potvrđenom NAFLDom. Analize krvi vršene su svaka tri meseca. Uvedena je kombinovana terapija pentoksifilinom u dozi od 400 mg 3×1 i metforminom u dozi od 500 mg 3×1, u trajanju od šest meseci za Nash grupu. Efekat lečenja procenjen je na osnovu biohemijskih parametara nakon terapije ovim medikamentima sa niskom cenom.

**Rezultati:**

Svih 33 pacijenta su završila 24 nedelje terapije. Uočeno je značajno poboljšanje (p<0,05) vrednosti medijane za sledeće parametre: nivoi mokraćne kiseline u serumu su se snizili za 51,0 mmol/L, kalcijum je snižen za 0,27 mmol/L, magnezijum je pokazao porast za 0,11 mmol/L. Poboljšana je insulinska rezistencija konstatovana kroz smanjenje HOMA-IR za 1,3. Zabeleženo je značajno smanjenje medijane enzima jetre: alanin aminotransferaze za 24,0 U/L, aspartat aminotransferaze za 9,1 U/L i gama glutamiltransferaze za 10,8 U/L.

**Zaključak:**

Pentoksifilin sa metforminom može predstavljati moguću terapijsku opciju u NASHu. Primećena je nova potencijalna korist primenjene terapije u poboljšanju funkcije jetre i smanjenju kardiovaskularnog rizika.

## Introduction

The epidemic extent of nonalcoholic fatty liver disease (NAFLD) reaching up to 30% of the general population, has become the most common chronic liver disease worldwide [1]. Nonalcoholic steatohepatitis (NASH), a progressive form of NAFLD, was first described almost 40 years ago, as a specific entity characterized by fatty changes with lobular hepatitis in patients with no history of alcoholism [2]. It has become increasingly identified since. Most studies are reporting a prevalence of biopsy-proven NASH of 3 to 5 percent among NAFLD patients [3] [4] [5]. This patient population exhibits increased rates of liver-related mortality as a result of cirrhosis and its complications, including hepatocellular carcinoma (HCC) [6]. The aforementioned remark is corresponding to the observation of NASH-associated cirrhosis becoming the most common cause of liver transplantation, exceeding hepatitis C virus and alcoholic related cirrhosis [7]. Moreover, given the lack of understanding of NAFLD prevalence added to NASH-associated cirrhosis, researchers note that the real disease burden may be significantly higher [8].

The progression of NAFLD is multifactorial, and there are currently no FDA-approved medications for the treatment of NASH [9]. One of the main obstacles remains in a fact that the NASH triggers have not been thoroughly recognized. Treatment options target different mechanisms responsible for the development of the disease. At this point, the beneficial effect to a certain extent has been shown by insulin-sensitizing agents, antioxidants, lipid-lowering agents, angiotensin receptor blockers, and probiotics. Promising results from the new drugs currently investigated in clinical trials, including obeticholic acid, liraglutide, elafibranor, and aramchol are being evaluated [10]. Lifestyle modification focused on diet and exercises continue to be the primary treatment [11]. The American Association for the Study of Liver Diseases practice guideline suggests the loss of at least 3-5% of body weight to improve steatosis, and up to 10% to improve necroinflammation [12]. However, most patients who ask medical advice have a long history of ineffective attempts at dietary and lifestyle changes [13]. Furthermore, many biochemical parameters, for instance, liver enzymes, platelets, albumin, and scoring systems based on aforementioned markers, have been used to investigate NAFLD [14] [15] [16].

Pentoxifylline (PTX), a phosphodiesterase inhibitor, although commonly used for peripheral vascular disease, is one of the antioxidants considered for NASH treatment. Besides its antioxidant activity and inhibition of proinflammatory cytokine production (tumor necrosis factor alpha - TNF-α), it exerts pleiotropic pharmacological effects [17]. Additionally, it has been implied that PTX reduces platelet-derived growth factor and tumor growth factor beta-1mediated fibrosis [18] [19]. A study by Zein et al. [20] noted that PTX markedly reduced steatosis, lobular inflammation, and liver fibrosis. Two meta-analyses revealed a decrease in liver enzymes and histological benefit in NASH patients after PTX therapy [21] [22]. Metformin (MET) exerts its effect on glucose homeostasis via adenosine monophosphate-activated protein kinase. It has proven certain benefits in NASH treatment. Some studies detected a reduction in aminotransferases levels in addition to the improvement of insulin resistance [23] [24], though the lack of histological response [25].

Acknowledging the complexity of the disease, it seems improbable that a single pharmacologic therapy will meet the requirements of the majority of this specific patient population. Hence, our study aimed to evaluate the efficacy of combination therapy of PTX and MET by monitoring biochemical parameters of patients with biopsy-diagnosed NASH.

## Materials and Methods

Data were collected prospectively from adult patients who attended a routine examination at the Clinic for Gastroenterology and Hepatology. Physical examination, abdominal ultrasound, laboratory testing, and liver biopsy were available for all patients. The first cohort included patients with biopsy-proven NASH. The second cohort consisted of age and gender-matched pairs with biopsy-confirmed NAFLD. Initially, all patients were instructed to follow the specific diet (*British Liver Trust*) and were counseled on other lifestyle modifications.

Exclusion criteria were other forms of primary hepatocellular liver diseases: hepatitis B, hepatitis C, autoimmune hepatitis, Wilson disease, alpha-1-antitrypsin deficiency, hemochromatosis, drug-induced liver disease. Patients on PTX or anti-diabetic drugs (insulin, biguanides, sulfonylureas, or thiazolidinediones), amiodarone, methotrexate, tamoxifen, corticosteroids in the previous six months as well as patients with a history of excess alcohol ingestion (consumption of alcohol 20 g per day) were also excluded from the study. Furthermore, this study dismissed patients with significant systemic or major illnesses (congestive heart failure, coronary artery disease, cerebrovascular disease, a pulmonary disease with hypoxia, renal failure, organ transplantation, severe psychiatric disease, malignancy) that might influence the treatment results.

### Abdominal ultrasonography

The abdominal ultrasound was performed using a Toshiba core vision with Doppler duplex convex probe 3.5 MHz. Corresponding to previous studies following criteria were applied: the diffuse hyperechoic echotexture, hepatorenal echo contrast in reference to the cortex of the right kidney, vascular blurring, and deep-echo attenuation [27] [28].

### Liver biopsy

Biopsies were performed for diagnostic purposes, during regular follow-up. Menghini liver biopsy needle was used. Histological criteria for steatohepatitis included: macrovesicular steatosis, hepatocellular injury, parenchymal and portal inflammation, fibrosis stage.

### Laboratory measurements

All patients fasted overnight before physicians saw them in the clinic. We determined their height and weight, and body mass index (BMI) expressed as a weight (kg) divided by the square of the height (m^2^).

Blood samples were collected under standard conditions between 7 and 8 am after 12h overnight fast. K_2_-EDTA-anticoagulated blood was used for performing complete blood count (Coulter LH 750, Beckman Coulter, Inc., USA). The analysis of hematological parameters were conducted immediately upon sampling, serum was separated by centrifugation, and analysis were performed. Biochemical parameters (glucose level, insulin, total cholesterol (TC) and triglyceride concentrations (TG), high-density lipoprotein cholesterol (HDL-C), low-density lipoprotein cholesterol (LDL-C), high sensitivity Creactive protein (hs-CRP), alanine aminotransferase (ALT), aspartate aminotransferase (AST), gamma-glutamyltransferase (GGT), serum uric acid levels (SUA), calcium, magnesium, vitamin B12, folate and homocysteine) were measured employing routine methods (Olympus System reagents using Olympus analyser AU 2700, Hamburg, Germany). Additionally, insulin resistance was assessed by the homeostasis model assessment (HOMA-IR) [26]. Blood tests were checked at baseline and every three months.

### Drug treatment

PTX at a dosage of 400 mg t.i.d. and MET at the dosage of 500 mg t.i.d. were introduced for six months in NASH cohort.

### Statistical analysis

Descriptive statistic was applied to provide basic characteristics of the study population. The one-sample Kolmogorov-Smirnov test was performed to determine if the data showed normal distribution. Mann Whitney test or T-test was used to compare parameters between cohorts. Measured parameters were further analyzed by Wilcoxon Signed Ranks Test in the treatment group to detect potential differences in data before and after medication use. A *p* value of less than 0.05 was considered statistically significant. The sample size was estimated based on Rosner calculation [29].

### Ethical considerations

The study was conducted in accordance with Guidelines for Good Clinical Practice, the Declaration of Helsinki, and local laws and regulations. The protocol was approved by the joint Research and Ethics Committee of the Clinical Centre of Serbia, Belgrade, filed under number 262/2. Written informed consent was obtained from all the participants in the study.

## Results

Patients in the NASH cohort and NAFLD cohort were similar in regards to their anthropometric and ultrasonographic characteristics but showed differences in some biochemical parameters (Table 1). We included 33 patients with NASH and 30 NAFLD patients.

**Table 1:** Baseline characteristics of the study population Data are given as mean ± standard deviation. BMI – body mass index, WBC – white blood cell count, Hgb – hemoglobin, MCH – mean corpuscular hemoglobin, MCHC – mean corpuscular hemoglobin concentration, RBC – red blood cells, PLT -platelets count; *CLCr – Creatinine Clearance calculated based on Cocroft-Gault formula; TC – total cholesterol, LDL – C - low density lipoprotein cholesterol, HDL-C – high-density lipoprotein cholesterol, TG – triglycerides, ALT – alanine aminotransferase, AST – aspartate aminotransferase, GGT – gamma-glutamyl transferase, CRP – C-reactive protein, SUA – serum uric acid, HbA1C – glycated haemoglobin, HOMA-IR – homeostasis model assessment of insulin resistance.

	NAFLD (n=30)	NASH (n=33)	*p* value
Age (years)	38.7 ± 8.1	40.8 ± 12.10	0.546
Male	20	20	/
Waist/Circumference	99.1 ± 11.9	103.1 ± 16.4	0.497
BMI (kg/m^2^)	27.3 ± 4.3	26.9 ± 3.5	0.844
Waist to hip ratio	0.9 ± 0.02	0.9 ± 0.02	0.965
WBC (109/L)	7.7 ± 2.3	8.7 ± 2.3	0.566
Hb (g/L)	136.0 ± 29.1	133.0 ± 21.2	0.307
MCH (pg)	28.8 ± 2.4	28.1 ± 2.0	0.778
MCHC (g/L)	35.8 ± 1.9	40.2 ± 2.9	0.452
RBC (10^12^/L)	4.9 ± 1.9	4.8 ± 0.9	0.664
PLT (10^9^/L)	275.9 ± 55.9	288.9 ± 43.9	0.778
CLCr* (mL/min)	98.3 ± 21.8	101.27 ± 25.6	0.405
TC (mmoL/L)	5.9 ± 1.4	5.8 ± 1.4	0.011
HDL-C (mmoL/L)	1.1 ± 0.4	1.0 ± 0.5	0.602
LDL-C (mmoL/L)	3.1 ± 1.3	3.2 ± 1.2	0.839
TG (mmoL/L)	1.7 ± 1.0	1.8 ± 1.2	0.944
Calcium (mmoL/L)	2.2 ± 0.15	2.5 ± 0.09	0.054
Magnesium (mmoL/L)	0.85 ± 0.15	0.67 ± 0.09	0.050
ALT (U/L)	38.5 ± 13.7	70.2 ± 35.1	0.001
AST (U/L)	25.5 ± 14.3	39.4 ± 18.2	0.001
GGT (U/L)	47.2 ± 24.8	54.8 ± 34.3	0.214
CRP (mg/L)	3.1 ± 2.4	3.2 ± 2.8	0.713
SUA (µmol/L)	346.3 ± 84.7	398.01± 108.5	0.041
HbA1c	5.5 ± 1.9	5.6 ± 1.3	0.338
HOMA-IR	4.7 ± 0.9	5.2 ± 1.7	0.010

### Adverse drug reactions

Patients reported the following adverse drug reactions (ADRs) during the first month of treatment: nausea (11%) and abdominal discomfort (14.9%). All ADRs were transient and mild; thus all patients included in the treatment group completed their 24 weeks medication regimen.

Most NASH patients lost weight, 3.1± 1.6 kg on average in observed six months period.

### Before and after drug treatment

We detected significantly different values after three and six months when compared to the baseline parameters before treatment in NASH cohort for the following parameters: SUA, ALT, AST, GGT, calcium, magnesium, and HOMA-IR, as presented in Table 2.

**Table 2:** Comparison of biochemical parameters after three and six months of treatment with baseline NASH Data are given as median with interquartile range. SUA – serum uric acid, ALT – alanine aminotransferase, AST – aspartate aminotransferase, GGT – gamma-glutamyl transferase, HOMA-IR – homeostasis model assessment of insulin resistance.

Parameters	NASH before	After 3 months	p-value	After 6 months	p-value
SUA (µmoL/L)	391.2 (339.0–482.3)	366.0 (327.9–416.1)	0.050	340.2 (291.2–390.2)	0.002
Calcium (mmoL/L)	2.50 (2.43–2.60)	2.35 (2.15–2.45)	0.040	2.23 (2.11–2.39)	0.002
Magnesium (mmoL/L)	0.66 (0.63–0.71)	0.72 (0.67–0.76)	0.030	0.77 (0.73–0.79)	0.004
ALT (U/L)	71.0 (46.1–92.0)	57.8 (39.0–63.2)	0.050	47.0 (39–59.9)	0.001
AST (U/L)	36.4 (25.9–55.5)	29.3 (19.0–47)	0.002	27.1 (17–41)	0.002
GGT (U/L)	49.80 (26.6–75.3)	45.6 (25.2–65.9)	0.050	39.0 (26–56)	0.005
HOMA-IR	5.0 (4.1–6.1)	4.20 (3.2–5.0)	0.009	3.7 (2.9–4.8)	0.001

In our study with given six months of NASH therapy, median SUA levels decreased by 51.0 µmol/L; serum calcium decreased by 0.27 mmoL/L, magne-sium showed an increase of 0.11 mmol/L. We detected a decrease in median values of ALT, AST and GGT by 24.0 U/L, 9.1 U/L, 10.8 U/L, respectively. Insulin resistance improved as a reduction of HOMA -IR by 1.3 was observed. Other monitored parameters (vitamin B12, homocysteine, folate) showed no statistically significant difference (p=0.106; 0.405; 0.203, respectively).

When the comparison of parameters between two cohorts, the NASH treatment group, and the NAFLD group were applied after the study period, we detected no significant difference for SUA, calcium, AST, and GGT.

**Figure 1:**
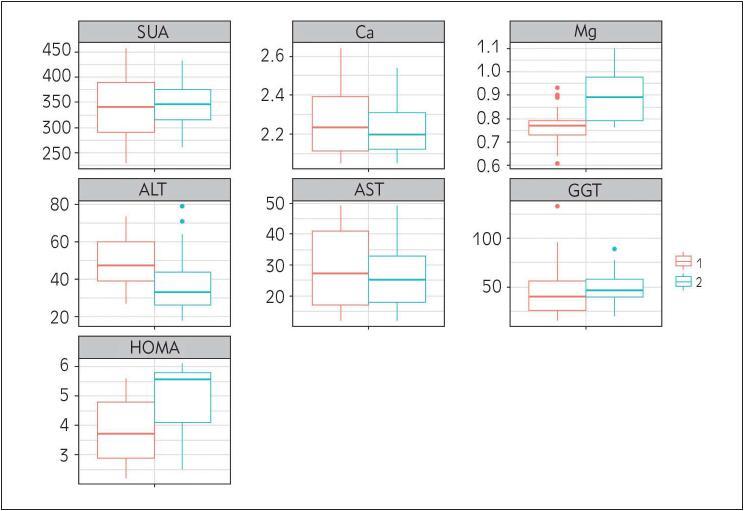
Diagram showing comparison of parameters in NASH cohort -1 (n=33) vs NAFLD cohort -2 (n=30) (after six months) SUA – serum uric acid, Ca – calcium, Mg – magnesium (1 vs 2, p<0.05), ALT – alanine aminotransferase (1 vs 2, p<0.05), AST – aspartate aminotransferase, GGT – gamma-glutamyl transferase), HOMA-IR – homeostasis model assessment of insulin resistance (1 vs 2, p<0.05).

However, magnesium, ALT, and HOMA-IR showed significant difference among observed cohorts after six months. In more details, ALT was still elevated in NASH group compared to NAFLD group (47.9 U/L vs. 37.7 U/L), magnesium reached the reference range in NASH treatment group but was lower than control group (0.77 mmoL/L vs. 0.90 mmoL/L), while HOMA-IR showed a decrease by 1.1 in NASH cohort when compared to NAFLD cohort (Figure 1).

## Discussion

It is previously demonstrated that PTX and MET have a positive effect when used as monotherapy in NASH outcomes [21] [25]. Thus, we aimed to explore the combined effect of the drugs, comprehending the multifactorial nature of NAFLD/NASH. Moreover, biopsy-confirmed NAFLD cohort served as a control group in our study, as all patients (NASH and NAFLD) were instructed to follow the specific diet and were counseled on other lifestyle modifications. NASH group additionally received combined drug treatment. To our knowledge, no studies of combination MET-PTX treatment in NASH patients have been published. Our data showed a substantial improvement in specific biochemical parameters after combined treatment in patients with biopsy-confirmed NASH even after three months. Observed parameters continued to improve after six months, with tolerable and mild adverse drug events during treatment. The NASH treatment group exhibited a similar profile in most studied parameters when compared to NAFLD control cohort after six months. Consequently, we suggest longer treatment duration.

There is a growing body of evidence implying that increased SUA has a role in NAFLD [30] [31]. Recently published studies have reported the association of uric acid levels and metabolic syndrome and NAFLD [32] [33] and furthermore recognized uric acid (and dietary fructose intake) as an independent factor associated with NASH [34]. Hyperuricemia has been linked to cardiovascular disease [35]. When NAFLD/NASH observed, the cardiovascular and not hepatic disease has been identified as the most common cause of death among this population [36]. Moreover, hyperuricemia has been related to insulin resistance, thus to hyperinsulinemia. This observation has been further explained by fructose intake, which may provoke metabolic syndrome and elevate uric acid, whereas lowering uric acid averts manifestation of metabolic syndrome [37]. Our data identified higher levels of SUA in the NASH group when compared to steatosis at a baseline, which confirms results from cited studies.

Interestingly, a reduction of SUA levels was detected after our drug intervention. This observation may be explained by PTX effect on minimizing liver damage while reducing SUA levels as suggested in lately published research [38]. Furthermore, PTX has been indicated to restore depressed cardiac output and improve hepatic perfusion and intestinal blood flow in case of hemorrhage and resuscitation [39]. We hypothesized that MET also adds to this effect through the reduction of insulin resistance, as we observed significantly lower HOMA-IR after treatment.

Notably, NASH patients had lower levels of magnesium at baseline similar to data from newly published research [40] but showed an increase in serum magnesium after MET-PTX therapy. These authors emphasized that lower serum magnesium concentration was independently related to NASH. Magnesium plays a role in insulin homeostasis, and evidence showed an inverse correlation between insulin resistance and magnesium intake, especially in overweight individuals [41]. Hence an explanation may be related to insulin resistance observed in our cohort as improving insulin resistance after given treatment is expected to elevate magnesium levels. This effect complies with MET mechanism of action. Consequently, our findings on improving magnesium levels with MET-PTX therapy add new insight into their potential benefit.

Moreover, a Korean population study by Shin et al. [42] revealed that elevated calcium and phosphorus levels were significantly related to NAFLD. Also, a study by Park et al. [43] disclosed a positive correlation between serum calcium and metabolic syndrome (while the negative correlation between magnesium and glucose). Our results go in line with these findings, as the NASH group showed higher calcium levels at a baseline. However, significantly lower calcium levels were achieved after six months of PTX-MET treatment. We argue that treatment added to reestablishing the balance between ions, again through improving insulin resistance.

As in previously published studies on patients on MET therapy [23] [24] [44], our findings after NASH treatment noted a significant decrease in liver aminotransferases (ALT, AST, and GGT) after six months of combination therapy. Furthermore, the HOMA-IR score reduced after treatment. Besides a well-documented effect of MET, PTX may support this improvement correspondingly to the results presented in a study by Van Wagner et al. [45].

Although in use for more than 50 years, MET and PTX's new beneficial properties are still being discovered. PTX is a methylxanthine, like caffeine, for which recent evidence indicates that increased caffeine intake may decrease liver fibrosis [46]. In addition, if basic direct therapy costs were observed, MET and PTX are low-cost medications. Having no approved conventional therapy for the treatment of NASH at this moment, health insurance not covering the expenses of NASH treatment in our healthcare system, hence patients are paying for the medicines by themselves. Therefore, efficacy, safety, and price of medicine should be considered when suggesting treatment options for NASH in developing countries. Despite the fact that this study is limited by the modest number of patients, it sheds light on additional use of easily available low-cost drugs, thus filling the knowledge gap between rigid clinical trials and reallife patient experience in actual clinical practice.

While the individual treatment of NASH including patient-tailored lifestyle based on pharmacogenomics expects to tackle the core of the disease [47], drugs that exert their mechanism of action based on currently available evidence, such as PTX and MET, may provide a possible treatment option.

## Conclusions

Treatment with PTX and MET of patients with NASH was well tolerated, with no clinically relevant side effects observed during our study. The results from this study imply some new potential benefit of PTX-MET treatment in improving liver function whilst decreasing cardiovascular risk in NASH patients.

*Author Contributions*: Conceptualization, D.C., S.VK., and M.C.; Methodology, D.C., V.D., and S.VK.; Formal Analysis, M.C. and S.VK.; Investigation, M.C., and N.B. Resources, B.O., V.D., and B.M.; Writing-Original Draft Preparation, M.C.; Writing-Review & Editing, M.C., D.C., S.VK. and B.M.; Supervision, S.VK. and D.C.

*Funding*: This research received no external funding.

## Conflict of interest statement

The authors stated that they have no conflicts of interest regarding the publication of this article.

## References

[1] Sayiner M, Koenig A, Henry L, Younossi Z M (2016). Epidemiology of Nonalcoholic Fatty Liver Disease and Nonalcoholic Steatohepatitis in the United States and the Rest of the World. Clin Liver Dis.

[2] Ludwig J, Viggiano T R, McGill D B, Oh B J (1980). Nonalcoholic steatohepatitis: Mayo Clinic experiences with a hitherto unnamed disease. Mayo Clin Proc.

[3] Lazo M, Hernaez R, Eberhardt M S, Bonekamp S, Kamel I, Guallar E, Koteish A, Brancati F L, Clark J M (2013). Prevalence of Nonalcoholic Fatty Liver Disease in the United States: The Third National Health and Nutrition Examination Survey, 1988-1994. Am J Epidemiol.

[4] Vernon G, Baranova A, Younossi Z M (2011). Systematic review: The epidemiology and natural history of non-alcoholic fatty liver disease and non-alcoholic steatohepatitis in adults. Aliment Pharmacol Ther.

[5] Williams C D, Stengel J, Asike M I, Torres D M, Shaw J, Contreras M, Landt C L, Harrison S A (2011). Prevalence of Nonalcoholic Fatty Liver Disease and Nonalcoholic Steatohepatitis Among a Largely Middle-Aged Population Utilizing Ultrasound and Liver Biopsy: A Prospective Study. Gastroenterology.

[6] Satapathy S K, Sanyal A J (2015). Epidemiology and Natural History of Nonalcoholic Fatty Liver Disease. Semin Liver Dis.

[7] Kabbany M N, Selvakumar P K C, Watt K K, Lopez R, Akras Z, Zein N, Carey W, Alkhouri N (2017). Prevalence of Nonalcoholic Steatohepatitis-Associated Cirrhosis in the United States: An Analysis of National Health and Nutrition Examination Survey Data. Am J Gastroenterol.

[8] Arrese M, Feldstein A (2017). Nash-related cirrhosis: An occult liver disease burden. Hepatology Communications.

[9] Noureddin M, Zhang A, Loomba R (2016). Promising therapies for treatment of nonalcoholic steatohepatitis. Expert Opin Emerg Drugs.

[10] Eshraghian A (2017). Current and emerging pharmacological therapy for non-alcoholic fatty liver disease. World J Gastroenterol.

[11] Promrat K, Kleiner D E, Niemeier H M, Jackvony E, Kearns M, Wands J R, Fava J L, Wing R R (2010). Randomized controlled trial testing the effects of weight loss on nonalcoholic steatohepatitis. Hepatology.

[12] Chalasani N, Younossi Z, Lavine J E, Diehl A M, Brunt E M, Cusi K, Charlton M, Sanyal A J (2012). The diagnosis and management of non-alcoholic fatty liver disease: Practice Guideline by the American Association for the Study of Liver Diseases, American College of Gastroenterology, and the American Gastroenterological Association. Hepatology.

[13] Dansinger M L, Tatsioni A, Wong J B, Chung M, Balk E M (2007). Meta-analysis: The Effect of Dietary Counseling for Weight Loss. Ann Intern Med.

[14] Angulo P, Hui J M, Marchesini G, Bugianesi E, George J, Farrell G C, Enders F, Saksena S, Burt A D, Bida J P, Lindor K, Sanderson S O, Lenzi M, Adams L A, Kench J (2007). The NAFLD fibrosis score: A noninvasive system that identifies liver fibrosis in patients with NAFLD. Hepatology.

[15] Harrison S A, Oliver D, Arnold H L, Gogia S, Neuschwander-Tetri B A (2008). Development and validation of a simple NAFLD clinical scoring system for identifying patients without advanced disease. Gut.

[16] Yilmaz Y, Yonal O, Kurt R, Bayrak M, Aktas B, Ozdogan O (2011). Noninvasive assessment of liver fibrosis with the aspartate transaminase to platelet ratio index (APRI): Usefulness in patients with chronic liver disease: APRI in chronic liver disease. Hepat Mon.

[17] Strieter R M, Remick D G, Ward P A, Spengler R N, Lynch J P 3rd, Larrick J (1988). Cellular and molecular regulation of tumor necrosis factor-alpha production by pentoxifylline. Biochem Biophys Res Commun.

[18] Shindel A W, Lin G, Ning H, Banie L, Huang Y C, Liu G (2010). Pentoxifylline attenuates transforming growth factor-beta1-stimulated collagen deposition and elastogenesis in human tunica albuginea-derived fibroblasts: Impact on extracellular matrix. J Sex Med.

[19] Verma-Gandhu M, Peterson M R, Peterson T C (2007). Effect of fetuin, a TGFbeta antagonist and pentoxifylline, a cytokine antagonist on hepatic stellate cell function and fibrotic parameters in fibrosis. Eur J Pharmacol.

[20] Zein C O, Yerian L M, Gogate P, Lopez R, Kirwan J P, Feldstein A E (2011). Pentoxifylline improves nonalcoholic steatohepatitis: A randomized placebo-controlled trial. Hepatology.

[21] Du J, Ma Y Y, Yu C H, Li Y M (2014). Effects of pentoxifylline on nonalcoholic fatty liver disease: A meta-analysis. World J Gastroenterol.

[22] Zeng T, Zhang C L, Zhao X L, Xie K Q (2014). Pentoxifylline for the treatment of nonalcoholic fatty liver disease: A meta-analysis of randomized double-blind, placebo-controlled studies. Eur J Gastroenterol Hepatol.

[23] Krakoff J, Clark J M, Crandall J P, Wilson C, Molitch M E, Brancati F L (2010). Effects of metformin and weight loss on serum alanine aminotransferase activity in the diabetes prevention program. Obesity (Silver Spring).

[24] Mazza A, Fruci B, Garinis G A, Giuliano S, Malaguarnera R, Belfiore A (2012). The role of metformin in the management of NAFLD.. Exp Diabetes Res.

[25] Li Y (2013). Metformin in non-alcoholic fatty liver disease: A systematic review and metaanalysis. Biomed Rep.

[26] Baumeister S E, Volzke H, Marschall P, John U, Schmidt C O, Flessa S (2008). Impact of fatty liver disease on health care utilization and costs in a general population: A 5-year observation. Gastroenterology.

[27] Sung K C, Ryan M C, Kim B S, Cho Y K, Kim B I, Reaven G M (2007). Relationships between estimates of adiposity, insulin resistance, and nonalcoholic fatty liver disease in a large group of nondiabetic Korean adults. Diabetes Care.

[28] Levy J C, Matthews D R, Hermans M P (1998). Correct homeostasis model assessment (HOMA) evaluation uses the computer program. Diabetes Care.

[29] Rosner B (1995). Fundamentals of Biostatistics.

[30] Liu C Q, He C M, Chen N, Wang D, Shi X, Liu Y (2016). Serum uric acid is independently and linearly associated with risk of nonalcoholic fatty liver disease in obese Chinese adults. Sci Rep.

[31] Sirota J C, Mcfann K, Targher G, Johnson R J, Chonchol M (2013). Elevated serum uric acid levels are associated with non-alcoholic fatty liver disease independently of metabolic syndrome features in the United States: Liver ultrasound data from the National Health and Nutrition Examination Survey. Metabolism.

[32] Kanbay M, Jensen T, Solak Y, Le M, Roncal-Jimenez C, Rivard C, Lanaspa M A, Nakagawa T, Johnson R J (2016). Uric acid in metabolic syndrome: From an innocent bystander to a central player. Eur J Intern Med.

[33] Lombardi R, Pisano G, Fargion S (2016). Role of Serum Uric Acid and Ferritin in the Development and Progression of NAFLD. International Journal of Molecular Sciences.

[34] Mosca A, Nobili V, de Vito R, Crudele A, Scorletti E, Villani A, Alisi A, Byrne C D (2017). Serum uric acid concentrations and fructose consumption are independently associated with Nash in children and adolescents. J Hepatol.

[35] Gagliardi A C M, Miname M H, Santos R D (2009). Uric acid: A marker of increased cardiovascular risk. Atherosclerosis.

[36] Targher G, Day C P, Bonora E (2010). Risk of Cardiovascular Disease in Patients with Nonalcoholic Fatty Liver Disease. N Engl J Med.

[37] Cirillo P, Sato W, Reungjui S, Heinig M, Gersch M, Sautin Y, Nakagawa T, Johnson R J (2006). Uric acid, the metabolic syndrome, and renal disease. J Am Soc Nephrol.

[38] Bektas S (2016). The effects of tadalafil and pentoxifylline on apoptosis and nitric oxide synthase in liver ischemia/reperfusion injury. Kaohsiung J Med Sci.

[39] Ribeiro E A, Poli-De-figueiredo L F, Vincenzi R, Galvao F H F, Margarido N, Rocha-E-silva M, Cruz R J (2013). Intraportal versus Systemic Pentoxifylline Infusion after Normothermic Liver Ischemia: Effects on Regional Blood Flow Redistribution and Hepatic Ischemia-Reperfusion Injury. HPB Surg.

[40] Eshraghian A, Nikeghbalian S, Geramizadeh B, Malek-Hosseini S A (2018). Serum magnesium concentration is independently associated with non-alcoholic fatty liver and non-alcoholic steatohepatitis. United European Gastroenterol J.

[41] Cahill F, Shahidi M, Shea J, Wadden D, Gulliver W, Randell E, Vasdev S, Sun G (2013). High Dietary Magnesium Intake Is Associated with Low Insulin Resistance in the Newfoundland Population. PLoS One.

[42] Shin J Y, Kim M J, Kim E S, Mo E Y, Moon S D, Han J H, Cha B Y (2015). Association between serum calcium and phosphorus concentrations with non-alcoholic fatty liver disease in Korean population. J Gastroenterol Hepatol.

[43] Park S H, Kim S K, Bae Y J (2012). Relationship Between Serum Calcium and Magnesium Concentrations and Metabolic Syndrome Diagnostic Components in Middle-Aged Korean Men. Biol Trace Elem Res.

[44] Idilman R, Mizrak D, Corapcioglu D, Bektas M, Doganay B, Sayki M, Coban S, Erden E, Soykan I, Emral R, Uysal A R, Ozden A (2008). Clinical trial: Insulin-sensitizing agents may reduce consequences of insulin resistance in individuals with non-alcoholic steatohepatitis. Aliment Pharmacol Ther.

[45] van Wagner L B, Koppe S W P, Brunt E M, Gottstein J, Gardikiotes K, Green R M, Rinella M E (2011). Pentoxifylline for the treatment of non-alcoholic steatohepatitis: A randomized controlled trial. Ann Hepatol.

[46] Modi A A, Feld J J, Park Y, Kleiner D E, Everhart J E, Liang T, Hoofnagle J H (2010). Increased caffeine consumption is associated with reduced hepatic fibrosis. Hepatology.

[47] Lorbek G, Urlep Ž, Rozman D (2016). Pharmacogenomic and personalized approaches to tackle nonalcoholic fatty liver disease. Pharmacogenomics.

